# Crystal structure and DFT study of a zinc xanthate complex

**DOI:** 10.1107/S2056989019013148

**Published:** 2019-10-03

**Authors:** Adnan M. Qadir, Sevgi Kansiz, Necmi Dege, Georgina M. Rosair, Igor O. Fritsky

**Affiliations:** a Department of Chemistry, College of Science, Salahaddin University, Erbil, Iraq; b Department of Physics, Faculty of Arts and Sciences, Ondokuz Mayıs University, 55139, Kurupelit, Samsun, Turkey; c Institute of Chemical Sciences, School of Engineering & Physical Sciences, Heriot-Watt University, Edinburgh, EH14 4AS, UK; d Taras Shevchenko National University of Kyiv, Department of Chemistry, 64, Vladimirska Str., Kiev 01601, Ukraine

**Keywords:** crystal structure, xanthate, zinc (II), DFT, mol­ecular electrostatic potential

## Abstract

In the title compound, bis­(2-meth­oxy­ethyl xanthato-κ*S*)(*N*,*N*,*N*′,*N*′-tetra­methyl­ethylenedi­amine-κ^2^ *N*,*N*′)zinc(II) acetone hemisolvate, [Zn(C_4_H_7_O_2_S_2_)_2_(C_6_H_16_N_2_)]·0.5C_3_H_6_O, the Zn^II^ ion is coordinated by two N atoms of the *N*,*N*,*N*′,*N*′-tetra­methyl­ethylenedi­amine ligand and two S atoms from two 2-meth­oxy­ethyl xanthate ligands. The amine ligand is disordered over two orientations and was modelled with refined occupancies of 0.538 (6) and 0.462 (6). The mol­ecular structure features two C—H⋯O and two C—H⋯S intra­molecular inter­actions. In the crystal, mol­ecules are linked by weak C—H⋯O and C—H⋯S hydrogen bonds, forming a three-dimensional supra­molecular architecture. The mol­ecular structure was optimized using density functional theory (DFT) at the B3LYP/6–311 G(d,p) level. The smallest HOMO–LUMO energy gap (3.19 eV) indicates the suitability of this crystal for optoelectronic applications. The mol­ecular electrostatic potential (MEP) further identifies the positive, negative and neutral electrostatic potential regions of the mol­ecules. Half a mol­ecule of disordered acetone was removed with the solvent-mask procedure in *OLEX2* [Dolomanov *et al.* (2009 ▸). *J. Appl. Cryst.* **42**, 339–341] and this contribition is included in the formula.

## Abstract 2

In the title compound, the Zn^II^ ion is coordinated by two N atoms of the *N*,*N*,*N*′,*N*′-tetra­methyl­ethylenedi­amine ligand and two S atoms from two 2-meth­oxy­ethyl xanthate ligands. Two C—H⋯O and two C—H⋯S intra­molecular inter­actions occur. In the crystal, mol­ecules are linked by C—H⋯O and C—H⋯S hydrogen bonds, forming a three-dimensional supra­molecular architecture.

## Chemical context   

Xanthates (di­thio­carbonates) are related to the di­thiol­ate family. Xanthate is a bidentate monoanionic sulfur–sulfur donor ligand. It stabilizes complexes of most of the transition elements and can bind metal centers in monodentate, isobidenate, anisobidenate or ionic modes. Xanthates have the ability to inhibit the replication of both RNA and DNA viruses *in vitro* (Friebolin *et al.*, 2005 ▸). They have been used as accelerators in the vulcanization of rubber (Gupta *et al.*, 2012 ▸), in cellulose synthesis (Tiravanti *et al.*, 1998 ▸), as collectors in the froth flotation of metal sulfide ores (Lee *et al.*, 2009 ▸) and as reagents for heavy-metal sedimentation in waste-water treatment (Chakraborty *et al.*, 2006 ▸). In our previous work, we prepared and structurally characterized nickel(II) and zinc(II) *n*-propylxanthate complexes containing *N*,*N*,*N*′,*N*′-tetra­methyl­ethylenedi­amine as a neutral ligand. Both complexes showed a distorted octa­hedral environment around the metal center (Qadir & Dege, 2019 ▸). In this paper, we report the synthesis and crystal structure of a zinc(II) 2-meth­oxy­ethylxanthate complex containing *N*,*N*,*N*′,*N′*-tetra­methyl­ethylenedi­amine, [Zn(S_2_COC_2_H_4_OCH_3_)_2_(tmeda)], which was investigated by a DFT study.
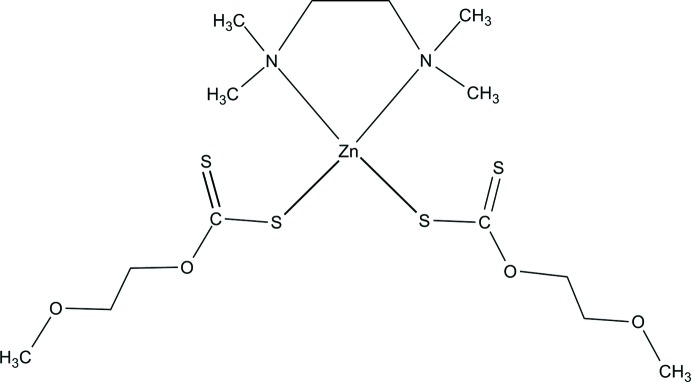


## Structural commentary   

**Figure 1:**
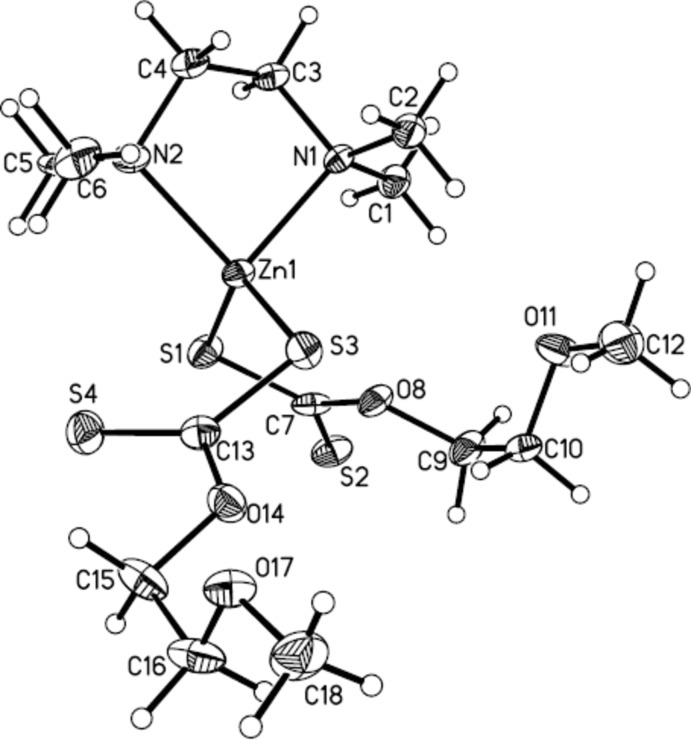
The mol­ecular structure of the title complex, with the atom labelling. Only the major component of the disordered amine ligand is shown. Displacement ellipsoids are drawn at the 50% probability level.

The mol­ecular structure of the title compound is illustrated in Fig. 1 ▸. The Zn^II^ ion is coordinated by two N atoms of the *N*,*N*,*N*′,*N′*-tetra­methyl­ethylenedi­amine mol­ecule and two S atoms from two 2-meth­oxy­ethylxanthate mol­ecules. The Zn1—N1, Zn1—N2, Zn1—S1 and Zn1—S3 bond lengths are 2.141 (5), 2.123 (5), 2.3107 (9) and 2.3050 (9) Å, respectively (Table 1 ▸). These bond distances are similar to those reported in the work of Cusack *et al.* (2004 ▸). The C7—O8 and C13—O14 bond lengths are similar [1.344 (3) and 1.346 (3) Å, respectively], while the C9—O8 and C15—O14 bonds are also not significantly different [1.454 (3) and 1.459 (3) Å, respectively]. In the same way, the C10—O11 [1.417 (3)] and C16—O17 [1.418 (4)] bond lengths are similar to each other. All of the C—O bonds show single-bond character. In the {S_2_C} section of the xanthate ligands, the carbon-to-sulfur S1 distance is 1.731 (3) Å, which is typical of a single bond whereas the carbon-to-sulfur S2 distance of 1.647 (3) Å is typical of a carbon-to-sulfur double bond. In the mol­ecule, weak C1—H1*C*⋯O8, C2*A*—H2*AB*⋯O11, C5*A*—H5*AA*⋯S1 and C6—H6*C*⋯S4 intra­molecular inter­actions are observed (Table 2 ▸).

## Supra­molecular features   

**Figure 2:**
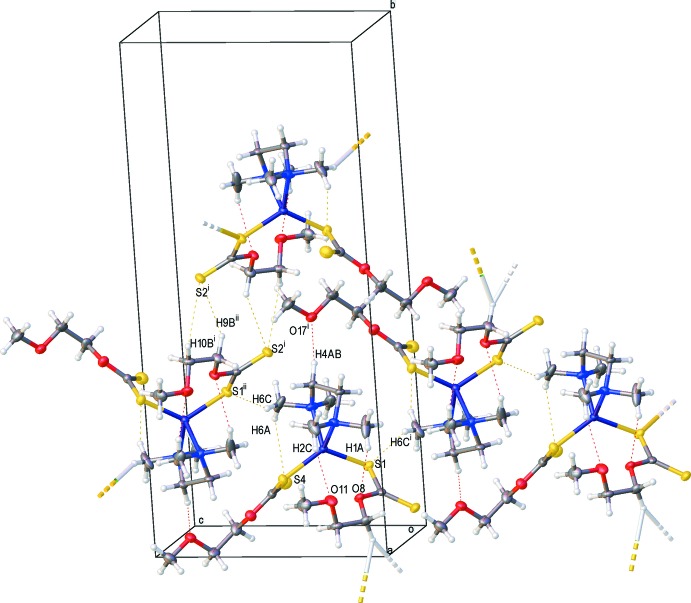
A view of the crystal packing of the title complex. Dashed lines denote the inter­molecular hydrogen bonds (Table 2 ▸). Symmetry codes: (i) *x* − 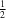, −*y* + 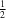, *z* − 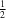; (ii) *x* + 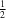, −*y* + 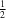, *z* + 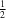.

The crystal packing of the title compound (Fig. 2 ▸) features inter­molecular hydrogen bonds (C6—H6*B*⋯O11^i^, C3*A*—H3*AB*⋯S2^ii^, C6*A*—H6*AA*⋯S1^ii^, C4*A*—H4*A*A⋯O17^iii^, C4*A*—H4*AB*⋯S3^iii^, C9—H9*A*⋯O17^iv^, C9—H9*B*⋯S2^v^ and C18—H18*B*⋯S2^vi^; symmetry codes as in Table 2 ▸), which connect the mol­ecules into a three-dimensional supra­molecular architecture.

## Database survey   

Previously reported complexes related to the title complex are [Cd(S_2_COCH_2_CH_2_OMe)_2_(bipy)] [CSD (Groom *et al.*, 2016 ▸) refcode BENDII; Chen *et al.*, 2002 ▸], [Ni(C_4_H_7_O_2_S_2_)_2_(C_6_H_16_N_2_)] (NADTAQ; Qadir, 2016 ▸), [Ni(moexa)_2_phen] (unsolvated form) and [Ni(moexa)_2_phen] (benzene solvate), moexa = *O*-methoxy­ethyl­xan­thato-*S*,*S*′ (with refcodes SICTUT and SICVAB, respectively; Edwards *et al.*, 1990*a* ▸), [Ni(moexa)_2_bpy]; forms I and II (with refcodes VETVIZ and VETVIZ01, respectively; Edwards *et al.*, 1990*b* ▸) and [Cd(S_2_COCH_2_CH_2_OCH_3_)_2_(4,7-Me_2_phen)] (WACPOG; Chen *et al.*, 2003 ▸). The Cd—S and Cd—N bond lengths range from 2.489 to 2.796 Å and 2.334 to 2.406 Å, respectively. Similarly, the Ni—S and Ni—N bond lengths range from 2.432 to 2.458 Å and 2.070 to 2.172 Å, respectively. In these complexes, compared with the Zn^II^ complex, the metal-to-ligand distances with *M*—S/N bond lengths follow the order Zn^II^ < Ni^II^ < Cd^II^ in the corresponding complexes.

## Frontier mol­ecular orbital analysis   

**Figure 3:**
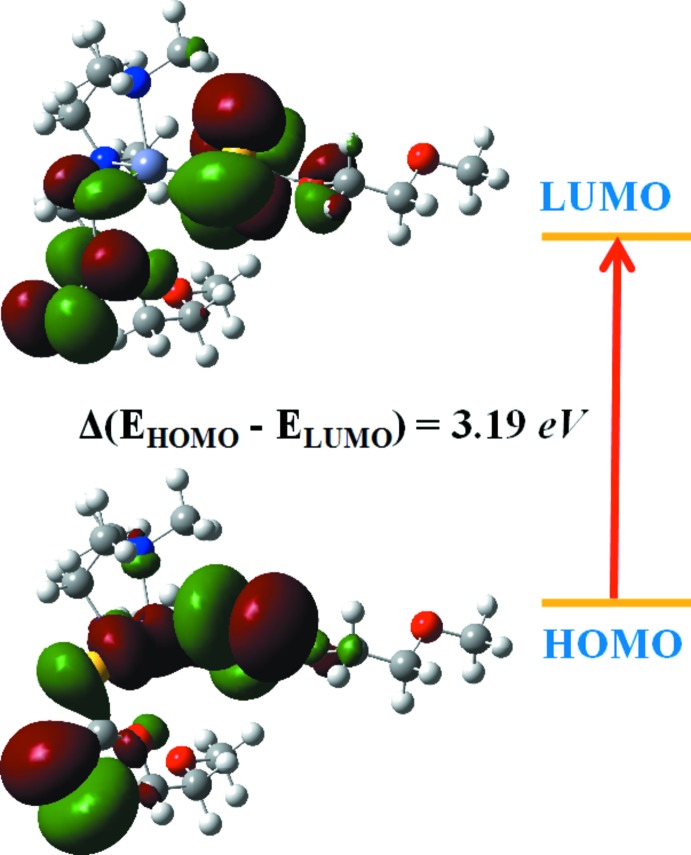
The electron distribution of the HOMO and LUMO energy levels of the title compound.

The highest occupied mol­ecular orbitals (HOMOs) and the lowest unoccupied mol­ecular orbitals (LUMOs) are named as frontier mol­ecular orbitals (FMOs). The FMOs play an important role in the optical and electric properties. The frontier orbital gap characterizes the chemical reactivity and the kinetic stability of the mol­ecule. A mol­ecule with a small frontier orbital gap is generally associated with a high chemical reactivity, low kinetic stability and is also termed a soft mol­ecule. The density functional theory (DFT) quantum-chemical calculations for the title compound were performed at the B3LYP/6–311 G(d,p) level (Becke, 1993 ▸) as implemented in *GAUSSIAN09* (Frisch *et al.*, 2009 ▸). Fig. 3 ▸ illustrates the HOMO and LUMO energy levels of the title compound. The small HOMO–LUMO energy gap (3.19 eV) in this compound indicates the chemical reactivity is strong and the kinetic stability is weak, which in turn increases the non-linear optical activity. As a result, with the small HOMO–LUMO energy gap, this compound could potentially be used in optoelectronic applications.

## Mol­ecular electrostatic potential (MEP)   

**Figure 4:**
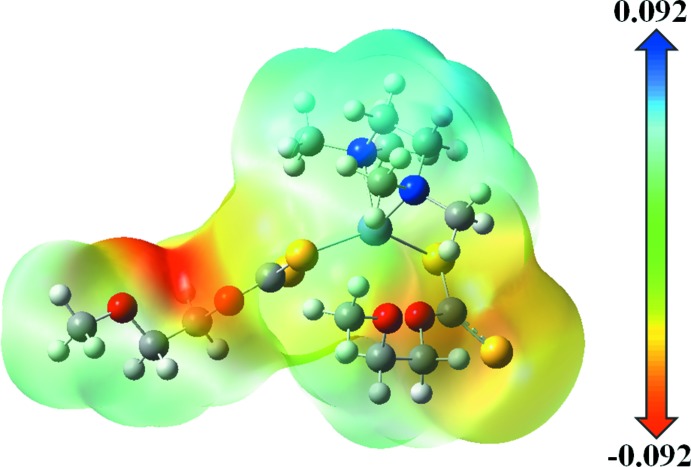
The total electron density three-dimensional surface mapped for the title compound with the electrostatic potential calculated at the B3LYP/6–311 G(d,p) level.

The MEP map of the title mol­ecule was calculated theoretic­ally at the B3LYP/6-311G(d,p) level of theory and is illustrated in Fig. 4 ▸. The blue-coloured region is electrophilic and electron poor, whereas the red colour indicates the nucleophilic region with rich electrons in the environment and provide information about inter­actions that can occur between mol­ecules (Tankov & Yankova, 2019 ▸). In the title compound, the reactive sites are localized near the C—O group: this is the region having the most negative potential spots (red colour), all over the oxygen atom because of the C—H⋯O inter­actions in the crystal structure. The negative potential value of −0.092 a.u. indicates the region that shows the strongest repulsion (electrophilic attack). In addition, the most positive region is located at the hydrogen atoms and shows the strongest attraction (nucleophilic attack) sites, which involve the *N*,*N*,*N*′,*N*′-tetra­methyl­ethylenedi­amine moiety.

## Synthesis and crystallization   

Tetra­methyl­ethylenedi­amine (10 mmol, 1.16 g) was added to a hot solution of Zn(CH_3_CO_2_)·2H_2_O (10 mmol, 2.20 g) in 2-meth­oxy­ethanol. A hot solution of potassium 2-meth­oxy­ethylxanthate (20 mmol, 3.81 g) in 2-meth­oxy­ethanol was added and the mixture was stirred for 30 min. Water was added to the mixture and a white precipitate was formed. The product was recrystallized from acetone.

## Refinement   

Crystal data, data collection and structure refinement details are summarized in Table 3 ▸. The C-bound H atoms were positioned geometrically and refined using a riding model, with C—H = 0.98 and 0.99 Å and with *U*_iso_(H) = 1.5*U*_eq_(C) for methyl H atoms and 1.2*U*_eq_(C) otherwise. All atoms of the amine ligand are disordered and were modelled as two orientations with relative occupancies of 0.538 (6) and 0.462 (6). The diffuse electron density of half an acetone solvent mol­ecule was removed with the solvent-mask procedure implemented in *OLEX2* (Dolomanov *et al.*, 2009 ▸). There are two voids of 122.4 Å^3^ in the unit cell and the electron count was 18.2 per void.

## Supplementary Material

Crystal structure: contains datablock(s) I. DOI: 10.1107/S2056989019013148/lh5921sup1.cif

Structure factors: contains datablock(s) I. DOI: 10.1107/S2056989019013148/lh5921Isup2.hkl

CCDC reference: 1420207

Additional supporting information:  crystallographic information; 3D view; checkCIF report

## Figures and Tables

**Table 1:** Selected geometric parameters (Å, °)

Zn1—S1	2.3107 (9)	S1—C7	1.731 (3)
Zn1—S3	2.3050 (9)	S2—C7	1.647 (3)
Zn1—N1	2.141 (5)	S3—C13	1.723 (3)
Zn1—N2	2.123 (5)	S4—C13	1.657 (3)
			
S3—Zn1—S1	125.54 (3)	N1—Zn1—S3	106.5 (2)
N1—Zn1—S1	105.2 (2)	N2—Zn1—N1	86.9 (2)

**Table 2:** Hydrogen-bond geometry (Å, °)

*D*—H⋯*A*	*D*—H	H⋯*A*	*D*⋯*A*	*D*—H⋯*A*
C1—H1*C*⋯O8	0.98	2.48	3.103 (7)	121
C2*A*—H2*AB*⋯O11	0.98	2.24	3.207 (13)	168
C5*A*—H5*AA*⋯S1	0.98	2.92	3.454 (16)	115
C6—H6*C*⋯S4	0.98	2.74	3.512 (13)	136
C6—H6*B*⋯O11^i^	0.98	2.54	3.321 (13)	136
C3*A*—H3*AB*⋯S2^ii^	0.99	2.81	3.483 (7)	125
C6*A*—H6*AA*⋯S1^ii^	0.98	2.84	3.764 (16)	158
C4*A*—H4*AA*⋯O17^iii^	0.99	2.44	3.380 (6)	159
C4*A*—H4*AB*⋯S3^iii^	0.99	2.81	3.774 (8)	164
C9—H9*A*⋯O17^iv^	0.99	2.61	3.415 (4)	138
C9—H9*B*⋯S2^v^	0.99	2.94	3.708 (3)	135
C18—H18*B*⋯S2^vi^	0.98	3.02	3.998 (3)	176

Symmetry codes: (i) 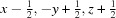; (ii) 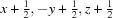; (iii) 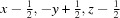; (iv) 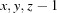; (v) 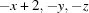; (vi) 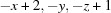.

**Table 3:** Experimental details

Crystal data	
Chemical formula	[Zn(C_4_H_7_O_2_S_2_)_2_(C_6_H_16_N_2_)]·0.5C_3_H_6_O
*M*_r_	513.05
Crystal system, space group	Monoclinic, *P*2_1_/*n*
Temperature (K)	100
*a*, *b*, *c* (Å)	9.604 (3), 22.785 (6), 11.374 (3)
β (°)	106.304 (12)
*V* (Å^3^)	2389.0 (12)
*Z*	4
Radiation type	Mo *K*α
μ (mm^−1^)	1.40
Crystal size (mm)	0.56 × 0.52 × 0.06

Data collection	
Diffractometer	Bruker APEXII CCD
Absorption correction	Multi-scan (*SADABS*; Bruker, 2009 ▸)
*T*_min_, *T*_max_	0.594, 0.746
No. of measured, independent and observed [*I* > 2σ(*I*)] reflections	35197, 5276, 3870
*R*_int_	0.054
(sin θ/λ)_max_ (Å^−1^)	0.650

Refinement	
*R*[*F* ^2^ > 2σ(*F* ^2^)], *wR*(*F* ^2^), *S*	0.040, 0.105, 1.06
No. of reflections	5276
No. of parameters	299
No. of restraints	244
H-atom treatment	H-atom parameters constrained
Δρ_max_, Δρ_min_ (e Å^−3^)	0.41, −0.69

Computer programs: *APEX2* and *SAINT* (Bruker, 2009 ▸), *SHELXT* (Sheldrick, 2015*a* ▸), *SHELXL* (Sheldrick, 2015*b* ▸) and *OLEX2* (Dolomanov *et al.*, 2009 ▸).

## supplementary crystallographic information

### Crystal data

**Table:**

[Zn(C_4_H_7_O_2_S_2_)_2_(C_6_H_16_N_2_)]·0.5C_3_H_6_O	*F*(000) = 1016
*M**_r_* = 513.05	*D*_x_ = 1.346 Mg m^−^^3^
Monoclinic, *P*2_1_/*n*	Mo *K*α radiation, λ = 0.71073 Å
*a* = 9.604 (3) Å	Cell parameters from 8223 reflections
*b* = 22.785 (6) Å	θ = 2.4–27.2°
*c* = 11.374 (3) Å	µ = 1.40 mm^−^^1^
β = 106.304 (12)°	*T* = 100 K
*V* = 2389.0 (12) Å^3^	Plate, colourless
*Z* = 4	0.56 × 0.52 × 0.06 mm

### Data collection

**Table:**

Bruker APEXII CCD diffractometer	3870 reflections with *I* > 2σ(*I*)
φ and ω scans	*R*_int_ = 0.054
Absorption correction: multi-scan (SADABS; Bruker, 2009)	θ_max_ = 27.5°, θ_min_ = 2.6°
*T*_min_ = 0.594, *T*_max_ = 0.746	*h* = −12→12
35197 measured reflections	*k* = −29→29
5276 independent reflections	*l* = −13→14

### Refinement

**Table:**

Refinement on *F*^2^	244 restraints
Least-squares matrix: full	Hydrogen site location: inferred from neighbouring sites
*R*[*F*^2^ > 2σ(*F*^2^)] = 0.040	H-atom parameters constrained
*wR*(*F*^2^) = 0.105	*w* = 1/[σ^2^(*F*_o_^2^) + (0.057*P*)^2^] where *P* = (*F*_o_^2^ + 2*F*_c_^2^)/3
*S* = 1.06	(Δ/σ)_max_ = 0.001
5276 reflections	Δρ_max_ = 0.41 e Å^−^^3^
299 parameters	Δρ_min_ = −0.68 e Å^−^^3^

### Special details

**Table:**

Geometry. All esds (except the esd in the dihedral angle between two l.s. planes) are estimated using the full covariance matrix. The cell esds are taken into account individually in the estimation of esds in distances, angles and torsion angles; correlations between esds in cell parameters are only used when they are defined by crystal symmetry. An approximate (isotropic) treatment of cell esds is used for estimating esds involving l.s. planes.

### Fractional atomic coordinates and isotropic or equivalent isotropic displacement parameters (Å^2^)

**Table:**

	*x*	*y*	*z*	*U*_iso_*/*U*_eq_	Occ. (<1)
Zn1	0.94679 (3)	0.20615 (2)	0.25390 (3)	0.01743 (10)	
S1	0.77332 (8)	0.16292 (3)	0.09443 (7)	0.02362 (17)	
S2	0.81448 (9)	0.08333 (3)	−0.10110 (7)	0.02709 (19)	
S3	1.11500 (8)	0.15676 (3)	0.40643 (6)	0.02205 (17)	
S4	0.82433 (9)	0.13279 (3)	0.44905 (8)	0.0312 (2)	
N1	1.0669 (9)	0.2630 (3)	0.1684 (7)	0.0187 (17)	0.538 (6)
N2	0.8649 (9)	0.2832 (2)	0.3157 (8)	0.0199 (19)	0.538 (6)
C1	1.0675 (9)	0.2455 (3)	0.0430 (7)	0.0234 (15)	0.538 (6)
H1A	0.9674	0.2412	−0.0084	0.035*	0.538 (6)
H1B	1.1167	0.2757	0.0081	0.035*	0.538 (6)
H1C	1.1187	0.2081	0.0465	0.035*	0.538 (6)
C2	1.2174 (11)	0.2697 (6)	0.2467 (12)	0.022 (2)	0.538 (6)
H2A	1.2162	0.2813	0.3294	0.034*	0.538 (6)
H2B	1.2689	0.2323	0.2505	0.034*	0.538 (6)
H2C	1.2669	0.3000	0.2121	0.034*	0.538 (6)
C3	0.9856 (7)	0.3192 (2)	0.1604 (6)	0.0229 (13)	0.538 (6)
H3A	0.8955	0.3170	0.0919	0.027*	0.538 (6)
H3B	1.0453	0.3517	0.1431	0.027*	0.538 (6)
C4	0.9477 (7)	0.3317 (2)	0.2780 (5)	0.0245 (13)	0.538 (6)
H4A	1.0384	0.3385	0.3442	0.029*	0.538 (6)
H4B	0.8894	0.3681	0.2679	0.029*	0.538 (6)
C5	0.7089 (10)	0.2896 (4)	0.2515 (12)	0.022 (2)	0.538 (6)
H5A	0.6950	0.2885	0.1628	0.033*	0.538 (6)
H5B	0.6548	0.2573	0.2750	0.033*	0.538 (6)
H5C	0.6736	0.3271	0.2740	0.033*	0.538 (6)
C6	0.883 (2)	0.2849 (6)	0.4502 (9)	0.029 (3)	0.538 (6)
H6A	0.9861	0.2808	0.4943	0.044*	0.538 (6)
H6B	0.8470	0.3225	0.4719	0.044*	0.538 (6)
H6C	0.8282	0.2527	0.4729	0.044*	0.538 (6)
N1A	1.0736 (10)	0.2669 (3)	0.1831 (8)	0.019 (2)	0.462 (6)
N2A	0.8391 (10)	0.2822 (3)	0.2930 (9)	0.020 (2)	0.462 (6)
C1A	1.0349 (10)	0.2672 (4)	0.0475 (7)	0.0209 (17)	0.462 (6)
H1AA	0.9303	0.2736	0.0142	0.031*	0.462 (6)
H1AB	1.0877	0.2988	0.0201	0.031*	0.462 (6)
H1AC	1.0611	0.2294	0.0184	0.031*	0.462 (6)
C2A	1.2323 (12)	0.2571 (7)	0.2278 (15)	0.026 (3)	0.462 (6)
H2AA	1.2624	0.2566	0.3175	0.039*	0.462 (6)
H2AB	1.2564	0.2194	0.1968	0.039*	0.462 (6)
H2AC	1.2830	0.2888	0.1985	0.039*	0.462 (6)
C3A	1.0409 (7)	0.3254 (3)	0.2283 (8)	0.0261 (15)	0.462 (6)
H3AA	1.0678	0.3568	0.1787	0.031*	0.462 (6)
H3AB	1.0991	0.3307	0.3145	0.031*	0.462 (6)
C4A	0.8818 (7)	0.3300 (3)	0.2199 (7)	0.0258 (15)	0.462 (6)
H4AA	0.8618	0.3687	0.2514	0.031*	0.462 (6)
H4AB	0.8237	0.3269	0.1332	0.031*	0.462 (6)
C5A	0.6793 (11)	0.2766 (5)	0.2581 (15)	0.022 (2)	0.462 (6)
H5AA	0.6435	0.2671	0.1708	0.033*	0.462 (6)
H5AB	0.6520	0.2452	0.3063	0.033*	0.462 (6)
H5AC	0.6365	0.3137	0.2741	0.033*	0.462 (6)
C6A	0.889 (2)	0.2966 (7)	0.4260 (10)	0.023 (3)	0.462 (6)
H6AA	0.9949	0.3006	0.4516	0.035*	0.462 (6)
H6AB	0.8448	0.3336	0.4409	0.035*	0.462 (6)
H6AC	0.8603	0.2651	0.4731	0.035*	0.462 (6)
C7	0.8778 (3)	0.11821 (10)	0.0299 (3)	0.0221 (6)	
O8	1.0160 (2)	0.11540 (7)	0.09989 (17)	0.0220 (4)	
C9	1.1153 (3)	0.07649 (11)	0.0618 (3)	0.0262 (7)	
H9A	1.1345	0.0914	−0.0139	0.031*	
H9B	1.0731	0.0367	0.0454	0.031*	
C10	1.2527 (3)	0.07470 (11)	0.1641 (3)	0.0246 (6)	
H10A	1.2301	0.0685	0.2429	0.030*	
H10B	1.3139	0.0417	0.1514	0.030*	
O11	1.3286 (2)	0.12830 (8)	0.1677 (2)	0.0318 (5)	
C12	1.4621 (3)	0.12837 (14)	0.2611 (3)	0.0380 (8)	
H12A	1.5113	0.1660	0.2607	0.057*	
H12B	1.4437	0.1226	0.3408	0.057*	
H12C	1.5237	0.0965	0.2463	0.057*	
C13	1.0016 (3)	0.12252 (11)	0.4785 (3)	0.0208 (6)	
O14	1.0774 (2)	0.08476 (8)	0.56361 (18)	0.0248 (4)	
C15	1.0045 (4)	0.05469 (13)	0.6430 (3)	0.0303 (7)	
H15A	0.9223	0.0311	0.5933	0.036*	
H15B	0.9664	0.0836	0.6911	0.036*	
C16	1.1136 (4)	0.01571 (12)	0.7265 (3)	0.0330 (8)	
H16A	1.0658	−0.0086	0.7762	0.040*	
H16B	1.1574	−0.0109	0.6779	0.040*	
O17	1.2226 (2)	0.05108 (8)	0.80437 (19)	0.0278 (5)	
C18	1.3393 (4)	0.01777 (13)	0.8787 (3)	0.0378 (8)	
H18A	1.4112	0.0443	0.9305	0.057*	
H18B	1.3026	−0.0089	0.9307	0.057*	
H18C	1.3847	−0.0050	0.8264	0.057*	

### Atomic displacement parameters (Å^2^)

**Table:**

	*U*^11^	*U*^22^	*U*^33^	*U*^12^	*U*^13^	*U*^23^
Zn1	0.01874 (19)	0.01410 (15)	0.01842 (19)	0.00001 (12)	0.00353 (13)	−0.00176 (11)
S1	0.0204 (4)	0.0212 (3)	0.0258 (4)	0.0008 (3)	0.0008 (3)	−0.0072 (3)
S2	0.0331 (5)	0.0199 (3)	0.0239 (4)	−0.0004 (3)	0.0008 (3)	−0.0061 (3)
S3	0.0203 (4)	0.0247 (3)	0.0203 (4)	0.0006 (3)	0.0042 (3)	0.0036 (3)
S4	0.0221 (4)	0.0326 (4)	0.0391 (5)	−0.0011 (3)	0.0089 (4)	−0.0034 (3)
N1	0.019 (3)	0.014 (3)	0.024 (3)	0.004 (2)	0.006 (3)	0.002 (2)
N2	0.023 (3)	0.016 (2)	0.018 (4)	−0.002 (2)	0.002 (3)	−0.003 (2)
C1	0.029 (4)	0.022 (4)	0.023 (3)	0.001 (3)	0.012 (3)	−0.003 (3)
C2	0.023 (4)	0.022 (5)	0.024 (5)	0.001 (3)	0.009 (3)	0.001 (3)
C3	0.027 (3)	0.013 (2)	0.029 (3)	0.003 (2)	0.009 (2)	0.003 (2)
C4	0.028 (3)	0.016 (2)	0.030 (3)	−0.002 (2)	0.009 (3)	−0.005 (2)
C5	0.019 (4)	0.011 (4)	0.034 (4)	−0.006 (3)	0.006 (3)	−0.003 (3)
C6	0.045 (5)	0.028 (5)	0.014 (4)	0.002 (4)	0.008 (4)	−0.008 (4)
N1A	0.022 (3)	0.016 (3)	0.020 (3)	−0.001 (3)	0.004 (3)	−0.001 (3)
N2A	0.025 (4)	0.016 (3)	0.016 (4)	−0.002 (2)	0.002 (3)	−0.009 (2)
C1A	0.024 (4)	0.024 (4)	0.016 (3)	−0.001 (3)	0.008 (3)	−0.005 (3)
C2A	0.013 (4)	0.033 (7)	0.030 (6)	−0.004 (4)	0.001 (4)	0.002 (4)
C3A	0.034 (3)	0.015 (2)	0.031 (3)	−0.002 (2)	0.010 (3)	−0.005 (2)
C4A	0.033 (3)	0.017 (2)	0.025 (3)	0.005 (3)	0.004 (3)	−0.002 (2)
C5A	0.016 (5)	0.013 (5)	0.033 (5)	−0.004 (3)	0.000 (4)	−0.006 (3)
C6A	0.023 (4)	0.033 (6)	0.014 (5)	−0.001 (4)	0.003 (4)	−0.005 (4)
C7	0.0281 (17)	0.0105 (11)	0.0239 (16)	−0.0027 (11)	0.0008 (13)	0.0009 (10)
O8	0.0218 (11)	0.0173 (9)	0.0240 (11)	0.0031 (7)	0.0019 (9)	−0.0058 (7)
C9	0.0274 (17)	0.0213 (13)	0.0292 (17)	0.0043 (12)	0.0070 (13)	−0.0070 (11)
C10	0.0270 (17)	0.0133 (12)	0.0343 (18)	0.0000 (11)	0.0097 (13)	−0.0023 (11)
O11	0.0291 (13)	0.0238 (10)	0.0375 (13)	−0.0099 (9)	0.0012 (10)	0.0063 (9)
C12	0.0253 (19)	0.0333 (17)	0.049 (2)	−0.0047 (13)	−0.0001 (16)	−0.0026 (14)
C13	0.0247 (16)	0.0187 (12)	0.0174 (15)	−0.0010 (11)	0.0033 (12)	−0.0016 (10)
O14	0.0255 (12)	0.0254 (10)	0.0253 (12)	−0.0016 (8)	0.0100 (9)	0.0046 (8)
C15	0.037 (2)	0.0319 (15)	0.0236 (18)	−0.0143 (13)	0.0105 (14)	0.0024 (12)
C16	0.052 (2)	0.0203 (14)	0.0284 (18)	−0.0091 (14)	0.0133 (15)	0.0034 (12)
O17	0.0337 (13)	0.0198 (9)	0.0277 (12)	0.0002 (8)	0.0051 (10)	0.0041 (8)
C18	0.044 (2)	0.0318 (16)	0.037 (2)	0.0101 (14)	0.0098 (16)	0.0124 (14)

### Geometric parameters (Å, º)

**Table:**

Zn1—S1	2.3107 (9)	C1A—H1AB	0.9800
Zn1—S3	2.3050 (9)	C1A—H1AC	0.9800
Zn1—N1	2.141 (5)	C2A—H2AA	0.9800
Zn1—N2	2.123 (5)	C2A—H2AB	0.9800
Zn1—N1A	2.144 (6)	C2A—H2AC	0.9800
Zn1—N2A	2.128 (6)	C3A—H3AA	0.9900
S1—C7	1.731 (3)	C3A—H3AB	0.9900
S2—C7	1.647 (3)	C3A—C4A	1.508 (7)
S3—C13	1.723 (3)	C4A—H4AA	0.9900
S4—C13	1.657 (3)	C4A—H4AB	0.9900
N1—C1	1.482 (6)	C5A—H5AA	0.9800
N1—C2	1.477 (7)	C5A—H5AB	0.9800
N1—C3	1.489 (6)	C5A—H5AC	0.9800
N2—C4	1.492 (6)	C6A—H6AA	0.9800
N2—C5	1.478 (7)	C6A—H6AB	0.9800
N2—C6	1.491 (7)	C6A—H6AC	0.9800
C1—H1A	0.9800	C7—O8	1.344 (3)
C1—H1B	0.9800	O8—C9	1.454 (3)
C1—H1C	0.9800	C9—H9A	0.9900
C2—H2A	0.9800	C9—H9B	0.9900
C2—H2B	0.9800	C9—C10	1.495 (4)
C2—H2C	0.9800	C10—H10A	0.9900
C3—H3A	0.9900	C10—H10B	0.9900
C3—H3B	0.9900	C10—O11	1.417 (3)
C3—C4	1.509 (6)	O11—C12	1.417 (4)
C4—H4A	0.9900	C12—H12A	0.9800
C4—H4B	0.9900	C12—H12B	0.9800
C5—H5A	0.9800	C12—H12C	0.9800
C5—H5B	0.9800	C13—O14	1.346 (3)
C5—H5C	0.9800	O14—C15	1.459 (3)
C6—H6A	0.9800	C15—H15A	0.9900
C6—H6B	0.9800	C15—H15B	0.9900
C6—H6C	0.9800	C15—C16	1.494 (4)
N1A—C1A	1.482 (7)	C16—H16A	0.9900
N1A—C2A	1.482 (7)	C16—H16B	0.9900
N1A—C3A	1.494 (7)	C16—O17	1.418 (4)
N2A—C4A	1.496 (7)	O17—C18	1.420 (4)
N2A—C5A	1.479 (7)	C18—H18A	0.9800
N2A—C6A	1.490 (7)	C18—H18B	0.9800
C1A—H1AA	0.9800	C18—H18C	0.9800
			
S3—Zn1—S1	125.54 (3)	H1AB—C1A—H1AC	109.5
N1—Zn1—S1	105.2 (2)	N1A—C2A—H2AA	109.5
N1—Zn1—S3	106.5 (2)	N1A—C2A—H2AB	109.5
N2—Zn1—S1	111.1 (2)	N1A—C2A—H2AC	109.5
N2—Zn1—S3	113.7 (2)	H2AA—C2A—H2AB	109.5
N2—Zn1—N1	86.9 (2)	H2AA—C2A—H2AC	109.5
N1A—Zn1—S1	109.9 (3)	H2AB—C2A—H2AC	109.5
N1A—Zn1—S3	104.2 (3)	N1A—C3A—H3AA	109.6
N2A—Zn1—S1	103.0 (3)	N1A—C3A—H3AB	109.6
N2A—Zn1—S3	121.3 (3)	N1A—C3A—C4A	110.4 (6)
N2A—Zn1—N1A	85.1 (3)	H3AA—C3A—H3AB	108.1
C7—S1—Zn1	101.98 (10)	C4A—C3A—H3AA	109.6
C13—S3—Zn1	100.13 (10)	C4A—C3A—H3AB	109.6
C1—N1—Zn1	115.0 (5)	N2A—C4A—C3A	110.2 (6)
C1—N1—C3	108.4 (5)	N2A—C4A—H4AA	109.6
C2—N1—Zn1	110.4 (8)	N2A—C4A—H4AB	109.6
C2—N1—C1	109.9 (7)	C3A—C4A—H4AA	109.6
C2—N1—C3	110.8 (7)	C3A—C4A—H4AB	109.6
C3—N1—Zn1	102.0 (4)	H4AA—C4A—H4AB	108.1
C4—N2—Zn1	103.9 (4)	N2A—C5A—H5AA	109.5
C5—N2—Zn1	109.6 (7)	N2A—C5A—H5AB	109.5
C5—N2—C4	109.5 (6)	N2A—C5A—H5AC	109.5
C5—N2—C6	108.4 (9)	H5AA—C5A—H5AB	109.5
C6—N2—Zn1	114.5 (7)	H5AA—C5A—H5AC	109.5
C6—N2—C4	110.9 (7)	H5AB—C5A—H5AC	109.5
N1—C1—H1A	109.5	N2A—C6A—H6AA	109.5
N1—C1—H1B	109.5	N2A—C6A—H6AB	109.5
N1—C1—H1C	109.5	N2A—C6A—H6AC	109.5
H1A—C1—H1B	109.5	H6AA—C6A—H6AB	109.5
H1A—C1—H1C	109.5	H6AA—C6A—H6AC	109.5
H1B—C1—H1C	109.5	H6AB—C6A—H6AC	109.5
N1—C2—H2A	109.5	S2—C7—S1	123.82 (18)
N1—C2—H2B	109.5	O8—C7—S1	111.76 (19)
N1—C2—H2C	109.5	O8—C7—S2	124.4 (2)
H2A—C2—H2B	109.5	C7—O8—C9	118.3 (2)
H2A—C2—H2C	109.5	O8—C9—H9A	110.3
H2B—C2—H2C	109.5	O8—C9—H9B	110.3
N1—C3—H3A	109.4	O8—C9—C10	107.1 (2)
N1—C3—H3B	109.4	H9A—C9—H9B	108.5
N1—C3—C4	111.1 (5)	C10—C9—H9A	110.3
H3A—C3—H3B	108.0	C10—C9—H9B	110.3
C4—C3—H3A	109.4	C9—C10—H10A	109.8
C4—C3—H3B	109.4	C9—C10—H10B	109.8
N2—C4—C3	113.3 (5)	H10A—C10—H10B	108.2
N2—C4—H4A	108.9	O11—C10—C9	109.5 (2)
N2—C4—H4B	108.9	O11—C10—H10A	109.8
C3—C4—H4A	108.9	O11—C10—H10B	109.8
C3—C4—H4B	108.9	C12—O11—C10	111.9 (2)
H4A—C4—H4B	107.7	O11—C12—H12A	109.5
N2—C5—H5A	109.5	O11—C12—H12B	109.5
N2—C5—H5B	109.5	O11—C12—H12C	109.5
N2—C5—H5C	109.5	H12A—C12—H12B	109.5
H5A—C5—H5B	109.5	H12A—C12—H12C	109.5
H5A—C5—H5C	109.5	H12B—C12—H12C	109.5
H5B—C5—H5C	109.5	S4—C13—S3	126.24 (16)
N2—C6—H6A	109.5	O14—C13—S3	110.2 (2)
N2—C6—H6B	109.5	O14—C13—S4	123.6 (2)
N2—C6—H6C	109.5	C13—O14—C15	119.2 (2)
H6A—C6—H6B	109.5	O14—C15—H15A	110.2
H6A—C6—H6C	109.5	O14—C15—H15B	110.2
H6B—C6—H6C	109.5	O14—C15—C16	107.5 (3)
C1A—N1A—Zn1	113.0 (6)	H15A—C15—H15B	108.5
C1A—N1A—C3A	109.8 (6)	C16—C15—H15A	110.2
C2A—N1A—Zn1	114.3 (9)	C16—C15—H15B	110.2
C2A—N1A—C1A	106.9 (8)	C15—C16—H16A	109.9
C2A—N1A—C3A	108.2 (7)	C15—C16—H16B	109.9
C3A—N1A—Zn1	104.5 (4)	H16A—C16—H16B	108.3
C4A—N2A—Zn1	104.6 (4)	O17—C16—C15	108.8 (2)
C5A—N2A—Zn1	113.7 (7)	O17—C16—H16A	109.9
C5A—N2A—C4A	109.8 (7)	O17—C16—H16B	109.9
C5A—N2A—C6A	107.8 (9)	C16—O17—C18	112.9 (2)
C6A—N2A—Zn1	110.8 (9)	O17—C18—H18A	109.5
C6A—N2A—C4A	110.1 (8)	O17—C18—H18B	109.5
N1A—C1A—H1AA	109.5	O17—C18—H18C	109.5
N1A—C1A—H1AB	109.5	H18A—C18—H18B	109.5
N1A—C1A—H1AC	109.5	H18A—C18—H18C	109.5
H1AA—C1A—H1AB	109.5	H18B—C18—H18C	109.5
H1AA—C1A—H1AC	109.5		
			
Zn1—S1—C7—S2	−170.71 (15)	C2—N1—C3—C4	−73.8 (9)
Zn1—S1—C7—O8	9.49 (19)	C5—N2—C4—C3	−84.1 (8)
Zn1—S3—C13—S4	−6.90 (19)	C6—N2—C4—C3	156.4 (9)
Zn1—S3—C13—O14	172.51 (16)	N1A—C3A—C4A—N2A	58.4 (9)
Zn1—N1—C3—C4	43.8 (6)	C1A—N1A—C3A—C4A	81.0 (8)
Zn1—N2—C4—C3	32.9 (7)	C2A—N1A—C3A—C4A	−162.6 (10)
Zn1—N1A—C3A—C4A	−40.5 (8)	C5A—N2A—C4A—C3A	−164.5 (9)
Zn1—N2A—C4A—C3A	−42.1 (8)	C6A—N2A—C4A—C3A	77.0 (10)
S1—C7—O8—C9	176.11 (18)	C7—O8—C9—C10	−171.4 (2)
S2—C7—O8—C9	−3.7 (3)	O8—C9—C10—O11	−73.4 (3)
S3—C13—O14—C15	174.79 (18)	C9—C10—O11—C12	−178.4 (2)
S4—C13—O14—C15	−5.8 (3)	C13—O14—C15—C16	179.5 (2)
N1—C3—C4—N2	−55.6 (8)	O14—C15—C16—O17	65.4 (3)
C1—N1—C3—C4	165.5 (6)	C15—C16—O17—C18	−173.9 (3)

### Hydrogen-bond geometry (Å, º)

**Table:**

*D*—H···*A*	*D*—H	H···*A*	*D*···*A*	*D*—H···*A*
C1—H1*C*···O8	0.98	2.48	3.103 (7)	121
C2*A*—H2*AB*···O11	0.98	2.24	3.207 (13)	168
C5*A*—H5*AA*···S1	0.98	2.92	3.454 (16)	115
C6—H6*C*···S4	0.98	2.74	3.512 (13)	136
C6—H6*B*···O11^i^	0.98	2.54	3.321 (13)	136
C3*A*—H3*AB*···S2^ii^	0.99	2.81	3.483 (7)	125
C6*A*—H6*AA*···S1^ii^	0.98	2.84	3.764 (16)	158
C4*A*—H4*AA*···O17^iii^	0.99	2.44	3.380 (6)	159
C4*A*—H4*AB*···S3^iii^	0.99	2.81	3.774 (8)	164
C9—H9*A*···O17^iv^	0.99	2.61	3.415 (4)	138
C9—H9*B*···S2^v^	0.99	2.94	3.708 (3)	135
C18—H18*B*···S2^vi^	0.98	3.02	3.998 (3)	176

Symmetry codes: (i) *x*−1/2, −*y*+1/2, *z*+1/2; (ii) *x*+1/2, −*y*+1/2, *z*+1/2; (iii) *x*−1/2, −*y*+1/2, *z*−1/2; (iv) *x*, *y*, *z*−1; (v) −*x*+2, −*y*, −*z*; (vi) −*x*+2, −*y*, −*z*+1.
